# Synergistic effect of puerarin and 5-fluorouracil on hepatocellular carcinoma

**DOI:** 10.3892/ol.2014.2534

**Published:** 2014-09-15

**Authors:** YAN-PING ZENG, ZI-RONG YANG, XU-FENG GUO, WANG JUN, WEI-GUO DONG

**Affiliations:** Department of Gastroenterology, Renmin Hospital of Wuhan University, Wuhan, Hubei 430062, P.R. China

**Keywords:** chemotherapy, 5-fluorouracil, hepatocellular carcinoma, puerarin

## Abstract

Hepatocellular carcinoma (HCC) is one of the most common types of human malignancy worldwide, which is becoming increasingly resistant to traditional drug treatments. Puerarin combined with 5-fluorouracil (5-FU) may be a useful treatment for liver cancer. The primary aim of the present study was to determine whether combined treatment with 5-FU and puerarin is more effective against the hepatocellular carcinoma (HCC) cell line, SMMC7721, than treatment with 5-FU or puerarin alone. The growth inhibition of SMMC7721 cells by puerarin or 5-FU alone or in combination was determined by the Cell Counting Kit-8 assay, *in vitro*. Apoptotic morphological features and the percentage of apoptotic cells were detected using Hoechst 33258 staining and an Annexin V/PI apoptosis kit, respectively. In addition, a tumor xenograft model was established in nude mice using SMMC7721 cells. Puerarin and 5-FU alone or in combination were injected into the mice, and the inhibition of tumor growth was evaluated by monitoring tumor volume and weight. Treatment with 6,400 or 640 μM 5-FU resulted in growth inhibition of 95.56±0.81 and 75.91±3.54%, respectively. The combination index values were <1 when the fraction of affected cells was between 0.2555 and 0.7420. Furthermore, the percentage of apoptotic cells was markedly increased in the combined treatment group when compared with that of the individual treatment groups, *in vitro* and *in vivo*. Individual treatment with puerarin resulted in a tumor volume inhibition rate (IR) of 70.58% and a tumor weight IR of 46.20%. Treatment with 5-FU was found to decrease the tumor volume by 76.26% and tumor weight by 49.86%. In the combined treatment group, the tumor volume and weight IRs were 93.11 and 75.21%, respectively. A marked increase in the inhibition of tumor growth and the number of apoptotic cells in response to combined treatment with puerarin and 5-FU was identified with no observed liver or renal toxicity. These results suggest that puerarin and 5-FU exhibit a synergistic treatment effect on the HCC SMMC7721 cell line.

## Introduction

Hepatocellular carcinoma (HCC) is the sixth most common type of human malignancy worldwide (1) and one of the most common types of malignant tumor in China. HCC is the third leading cause of cancer-related mortality worldwide (2,3) and the second leading cause of cancer-related mortality in China. The annual incidence of HCC is increasing (4,5) and, therefore, it presents a significant threat to human health. Chemotherapy may be used to treat HCC; however, traditional systemic chemotherapy exhibits a low curative rate for liver cancer due to its high number of toxic effects, and is therefore not widely accepted. Recently, treatments combining several chemotherapeutic or chemopreventive agents have been used as they not only enhance the treatment effect but also reduce drug toxicity.

The compound 5-fluorouracil (5-FU) is one of the most commonly used chemotherapeutic drugs for liver, colorectal and gastric cancers in clinical practice (6). In addition, 5-FU is commonly used in advanced-stage HCC chemotherapy, alone or in combination with other drugs. Resistance to 5-FU is a key cause of chemotherapy failure in advanced-stage HCC (7) and, therefore, it is important to identify novel chemotherapeutic agents with high therapeutic efficacy. The combination of novel chemotherapeutic agents with existing drugs may enhance the efficacy of liver cancer therapy.

*Radix puerariae* (the root of the kudzu plant, *Pueraria lobata*) is one of the most popular traditional Chinese medicines. Puerarin (daidzein-8-C-glucoside) and daidzein (daidzein-7-O-glucoside) are the major isoflavones of the kudzu root (8). Previous studies have suggested that puerarin may exhibit anticarcinogenic effects (9–16). In addition, one study has suggested that puerarin may act as a sensitizer to enhance the inhibitory effect of *Bulbophyllum* extract on HCC proliferation (17).

The aim of the present study was to investigate the effects of the combined treatment with puerarin and 5-FU on HCC *in vitro* and *in vivo*.

## Materials and methods

### Chemicals and reagents

Puerarin (P5555) was obtained from Sigma-Aldrich (St. Louis, MO, USA) with a purity of 98%, as assessed by reverse-phase high-performance liquid chromatography. The puerarin was stored as a 100-mM stock solution in dimethyl sulfoxide at −20°C and diluted with serum-free culture medium for use in experiments. The compound 5-FU was obtained from Sigma-Aldrich (F6627) and diluted to various concentrations in serum-free culture medium. The Cell Counting Kit-8 (CCK-8) was obtained from Dojindo Molecular Technologies, Inc. (Kumamato, Japan).

The Annexin V-fluorescein isothiocyanate (FITC)/propidium iodide (PI) kit was obtained from Beijing MultiSciences Biotech Co., Ltd (Beijing, China) and the Hoechst staining kit was obtained from the Beyotime Institute of Biotechnology (Shanghai, China).

### Cell line and cell culture

The HCC SMMC7721 cell line was obtained from the China Center for Type Culture Collection (Wuhan, China). The cells were cultured in Dulbecco’s modified Eagle’s medium (Gibco-BRL, Gaithersburg, MD, USA) supplemented with 10% fetal bovine serum (FBS; Gibco-BRL), 50 mg/ml streptomycin (Beyotime Institute of Biotechnology), 50 IU/ml penicillin (Beyotime Institute of Biotechnology), and 2 mM glutamine (Beyotime Institute of Biotechnology) in a humidified atmosphere of 5% CO_2_ at 37°C.

### Cell growth inhibition studies

The inhibitory effect of puerarin on SMMC7721 cell growth *in vitro* was determined using CCK-8 dye, which is only absorbed by living cells. Briefly, 5,000 cells/well were seeded into 96-well microtiter plates. Following exposure to puerarin (400, 800, 1,600, 3,200 or 6,400 μM), 5-FU (40, 80, 160, 320 or 640 μM) or puerarin and 5-FU (400:40; 800:80; 1600:60; 3200:320 μM, respectively) for 48 h, 10 μl CCK-8 solution was added to each well, and the plates were incubated for an additional 2 h (at 37°C with 5% CO_2_). The optical density was then determined at a wavelength of 450 nm using a microplate reader (iMark; Bio-Rad, Hercules, CA, USA). Each experiment was performed in triplicate and the results were presented as the inhibition rate (IR), which was calculated using the following formula: IR (%) = [(A-B)/A] × 100, where A and B are the absorbance of the control and sample groups following 48 h of incubation, respectively.

### Evaluation of the combined effects of puerarin and 5-FU

The following equation (18,19) was used to evaluate the nature of the interaction between puerarin and 5-FU: D = D_m_[fa/(1 - fa)]^1/m^, where D is the dose, D_m_ is the dose required to produce the median effect (analogous to the IC_50_), fa is the fraction of the system affected by D, and m is a Hill-type coefficient signifying the sigmoidicity of the dose-effect curve. The combination index (CI) values were obtained using Biosoft CalcuSyn software (Biosoft, Cambridge, UK) written in BASIC for automatic graphing of the CI with respect to fa, to determine whether the two drugs were non-exclusive. Synergism was indicated by a CI value of <1, summation by a CI value equal to 1 and antagonism by a CI value of >1.

### Cell apoptosis assay

Apoptotic cells were detected by Hoechst 33258 staining as follows. Following exposure to puerarin (1,600 μM), 5-FU (160 μM) or puerarin and 5-FU for 48 h, the SMMC7721 cells were incubated with 20 μM Hoechst 33258 (Beyotime Institute of Biotechnology) for 10 min at room temperature. The cells were then washed twice with phosphate-buffered saline (PBS) and examined under a fluorescence microscope (BX53F; Olympus, Tokoyo, Japan). The apoptotic cells were identified by Hoechst 33258 staining of the condensed chromatin and nuclear fragments. A total of 10 random fields were counted for each sample.

### Annexin V/PI staining

An Annexin V/PI apoptosis kit was used according to the manufacturer’s instructions to quantify the percentage of cells undergoing apoptosis. Firstly, SMMC7721 cells were incubated for 48 h with puerarin (1,600 μM) or 5-FU (160 μM) alone or in combination. Next, the cells were washed twice with cold PBS and resuspended in binding buffer at a concentration of 1×10^6^ cells/μl. Then, 5 μl of Annexin V-FITC and 10 μl PI were added, and the cells were incubated for 5 min at room temperature in the dark. Following incubation, 200 μl of binding buffer was added and the cells were analyzed immediately by flow cytometry (FACSAriaIII; BD Biosciences, Franklin Lakes, NJ, USA). The flow cytometry analysis was performed using the Cell Quest software (BD Biosciences). The Annexin V^+^/PI^−^ cells were identified as apoptotic cells, and the Annexin V^−^/PI^+^ cells were identified as necrotic cells (20). The entire procedure was repeated three times for each sample.

### Xenograft tumor model

All animal procedures, which complied with the National Institutes of Health Guide for the Care and Use of Laboratory Animals (21), were approved by the Committee on Animal Experimentation of Wuhan University (Wuhan, China). Four- to six-week-old male BALB/c-nu/nu nude mice were purchased from the Center for Experimental Animals of Wuhan University (Wuhan, China). All mice weighed 16–18 g and were bred in autoclaved, filter-top, microisolator cages, which were kept in an isolator unit with filtered air. The mice had access to water and food *ad libitum*. The human HCC SMMC7721 cell line used for inoculation was cultured as previously described. The mice were inoculated subcutaneously with 1×10^7^ SMMC7721 cells per mouse and the tumor sizes were measured using micrometer calipers. The mice were randomly divided into the following four groups, with six mice per group: Saline tumor control, puerarin (50 mg/kg/day), 5-FU (12 mg/kg/day), and puerarin (50 mg/kg/day) in combination with 5-FU (12 mg/kg/day).

### Measuring tumor volume and weight

The tumor volume (TV) was calculated using the following formula: TV (mm^3^) = d^2^x(D/2), where d and D are the shortest and longest diameters, respectively. The tumor size was measured with calipers every three days. The mice were sacrificed by extracting the eyeball at the end of four weeks and the tumor xenografts were removed, weighed and measured for additional analyses.

### Terminal deoxynucleotidyl transferase dUTP nick end labeling assay

Sections of each tumor xenograft were fixed in 4% formaldehyde, dehydrated with an ethanol gradient, embedded in paraffin, dewaxed and rehydrated with a decreasing ethanol gradient (100, 95, 90, 80 and 70%), according to standard instructions. An *in situ* apoptosis detection kit (Roche Molecular Systems Inc., Branchburg, NJ, USA) was used to detect apoptosis. All procedures were performed according to the manufacturer’s instructions. The specimens were incubated with proteinase K [15 μg/ml in 10 mM Tris/HCl (pH 7.5)] for 20 min at room temperature after being dewaxed and rehydrated. Next, the specimens were rinsed with 3% H_2_O_2_, and incubated with equilibration buffer and terminal deoxynucleotidyl transferase (Beyotime Institute of Biotechnology). The specimens were then incubated with an anti-digoxigenin-peroxidase conjugate. Finally, the 3,3′-diaminobenzidine substrate was added to react with the peroxidase and the specimens were counterstained with hematoxylin, mounted and analyzed using a light microscope (BX53F).

### Evaluation of side effects

Following the completion of the study, blood was collected from the nude mice by cardiac puncture using heparin-rinsed 5-ml syringes and 22-gauge needles. Alanine aminotransferase (ALT), aspartate aminotransferase (AST), blood urea nitrogen (BUN) and serum creatinine (Cr) levels were measured to evaluate liver and renal injury using an Olympus AU5400 Immuno Analyzer (Olympus). All mice were sacrificed and dissected following the completion of the study. In addition, metastasis, hemorrhage and liver and kidney morphology were assessed by an observer blinded to the treatment groups. This study was approved by the Institutional Review Board of Renmin Hospital at Wuhan University.

### Statistical analysis

Statistical analyses were performed using SPSS 17.0 software (SPSS, Inc., Chicago, IL, USA). All data are presented as the mean ± standard deviation (SD). The means of the different groups were compared using non-parametric analysis (Mann-Whitney rank-sum test) and P<0.05 was considered to indicate a statistically significant difference.

## Results

### Effect of single drug exposure on the growth of the HCC SMMC7721 cell line

**Figure 1:**
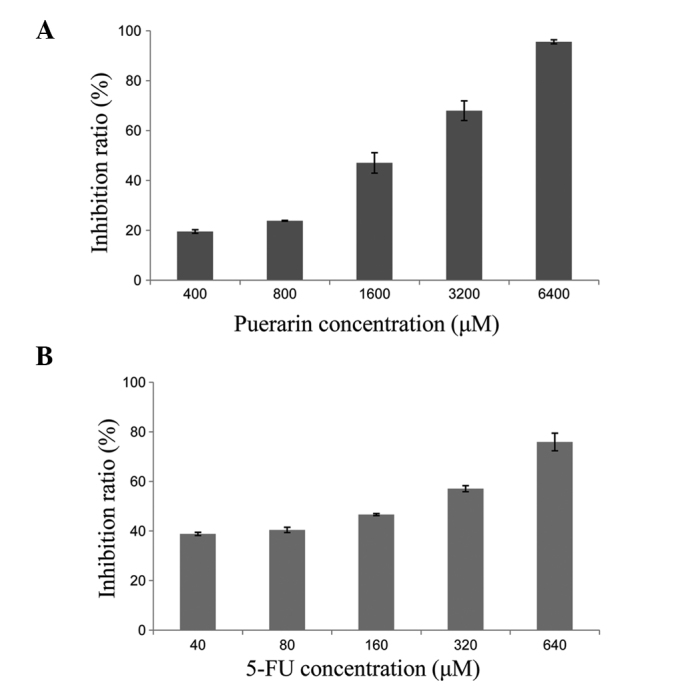
Effect of puerarin or 5-FU alone on SMMC7721 cell proliferation. (A) The proliferation of SMMC7721 cells was inhibited by puerarin in a dose-dependent manner (P<0.01). (B) Similar results were obtained for 5-FU (P<0.01). 5-FU, 5-fluorouracil.

The inhibition of SMMC7721 proliferation by puerarin and 5-FU was assessed following 48 h of drug exposure and 24 h of culture in drug-free medium. After 48 h of treatment, *in vitro* SMMC7721 cell growth was significantly inhibited in a dose-dependent manner (P<0.01; Fig. 1). The mean ± SD rate of inhibition by puerarin was 19.51±0.72% at a concentration of 400 μM, and 95.56±0.81% at a concentration of 6,400 μM. The rate of growth inhibition by 5-FU at 40 μM was 38.83±0.63%, while the rate was 75.91±3.54% at 640 μM.

### Combined effect of puerarin and 5-FU on SMMC7721 cell growth

**Figure 2:**
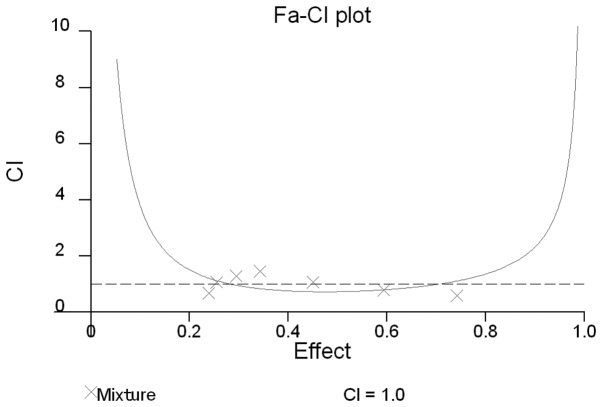
Combined effects of puerarin and 5-FU on the hepatocellular carcinoma cell line, SMMC7721. The CI values were determined using the method described by Chou and Talalay (18,19). CI=1, cumulative effect; CI<1, synergistic effect; and CI>1, antagonistic effect. The CI values for puerarin and 5-FU were <1 when the fractions affected were between 0.2555 and 0.7420. Thus, at specific concentrations, puerarin and 5-FU exhibit a synergistic inhibitory effect on the proliferation of SMMC7721 cells. 5-FU, 5-fluorouracil; CI, combination index.

The SMMC7721 cells were exposed to the two drugs in combination at a fixed molar ratio (puerarin to 5-FU, 10:1) for 48 h. The effect on cell proliferation was then assessed by CCK-8 assay and the rate of growth inhibition was calculated according to the method used by Chou and Talalay (18,19). All experiments were repeated in triplicate. The CI values were <1 when the fraction of affected cells was between 0.2555 and 0.7420 (Fig. 2).

### Apoptosis induced by puerarin and 5-FU

**Figure 3:**
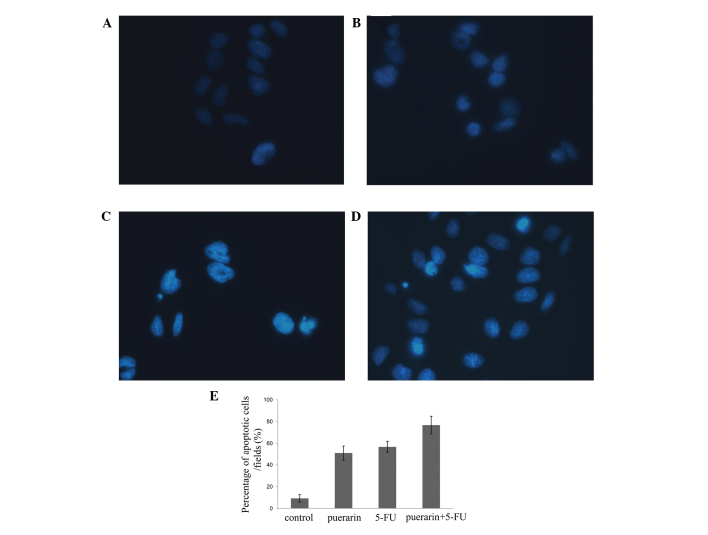
Detection of apoptosis-related morphological changes by Hoechst 33258 staining in SMMC7721 cells. (A) Control cells and cells exposed to (B) puerarin (1,600 μM), (C) 5-FU (160 μM) or (D) puerarin and 5-FU for 48 h were then incubated with 20 μM Hoechst 33258 for 10 min at room temperature. Apoptotic cells were identified by chromatin condensation and nuclear fragment staining. (E) The apoptotic cell rate in combined treatment group was higher than that of the puerarin and 5FU alone. Data are presented as the mean ± SD. ^*^P<0.05, vs. control cells.

Morphological changes in the SMMC7721 cells indicating apoptosis were detected by Hoechst 33258 staining. The classical characteristics of apoptotic cells are nuclear fragmentation and chromatin condensation. Distinct chromatin condensation and nuclear fragmentation were identified in the treatment groups, while the nuclei of the control cells stained a weak homogeneous blue (Fig. 3).

**Figure 4:**
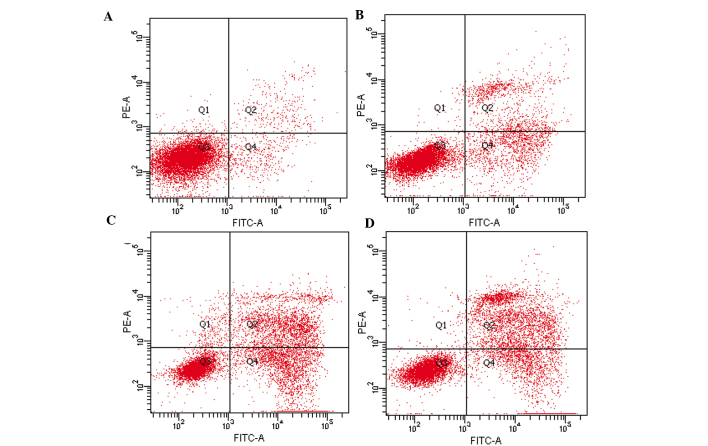
An Annexin V/PI apoptosis kit was used to quantify the percentage of cells undergoing apoptosis (X axis, Annexin V and Y axis, PI). Puerarin and 5-FU treatment alone and in combination induce apoptosis in SMMC7721 cells. SMMC7721 cells in (A) the control group and treated with (B) puerarin (1,600 μM), (C) 5-FU (160 μM) or (D) the two drugs in combination for 48 h. The Annexin V^+^/PI^−^ cells were apoptotic cells, and the Annexin V^−^/PI^+^ were necrotic cells. The percentages indicate the proportion of apoptotic cells. Puerarin alone or in combination with 5-FU was found to significantly induce apoptosis compared with the control group (P<0.01), and the degree of apoptosis induced by the combined treatment was greater than the effect of puerarin or 5-FU alone (P<0.01).

An Annexin V/PI apoptosis kit was used to quantify the percentage of cells undergoing apoptosis. The proportion of Annexin V-positive/PI-negative cells increased progressively over 48 h in the SMMC7721 cells incubated with low concentrations of puerarin (1,600 μM) and 5-FU (160 μM) (Fig. 4). The cells treated with puerarin or 5-FU demonstrated a significantly higher rate of apoptosis when compared with that of the control group (P<0.01; Fig. 4), and the rate of apoptosis was significantly higher in the combined treatment group when compared with that of the individual treatment groups (P<0.01; Fig. 4).

### Effect of puerarin and 5-FU on tumor development in vivo Xenograft tumor model and inhibition of SMMC7721 tumor growth

The effect of puerarin and 5-FU on the growth of primary tumor xenografts was investigated in nude mice. None of the mice succumbed to the disease during the study, and 18 mice successfully grew tumor xenografts. The mice were randomly divided into three groups as previously described, and a saline-only control group was included. No significant differences were identified in the tumor sizes among the four groups at the start of treatment. The mice were treated with puerarin, 5-FU, puerarin and 5-FU in combination, or saline only. Puerarin or 5-FU alone, as well as the combined treatment with the two drugs, significantly inhibited the SMMC7721 tumor growth rate (as determined by tumor volume and weight) when compared with that of the control group (P<0.05; Tables I and II).

### Detection of apoptotic cells in xenograft tumor tissue

**Figure 5:**
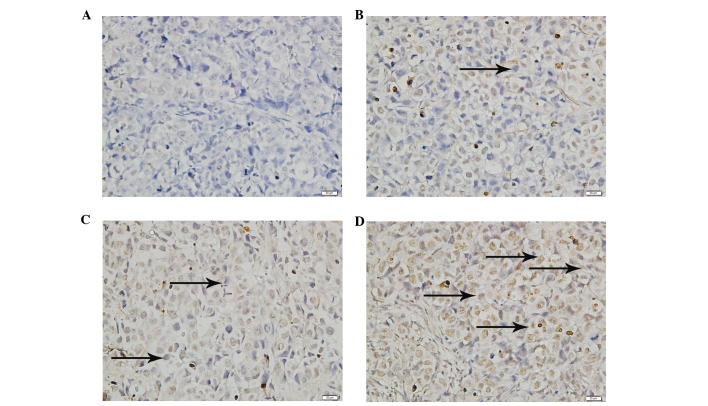
Detection of apoptotic cells in xenograft tumor tissue using terminal deoxynucleotidyl transferase dUTP nick end labeling assay. (A) Tissues from the tumors of the control group and tumors treated with (B) puerarin (50 mg/kg/day), (C) 5-FU (10 mg/kg/day) and (D) 5-FU (10 mg/kg/day) + puerarin (50 mg/kg/day). 5-FU, 5-fluorouracil.

The TUNEL assay demonstrated that significant cell death had occurred in the tumor masses from the treatment groups when compared with that of the saline-only control group (P<0.01; Fig. 5); however, the degree of apoptosis differed in each group. The tumors from the combined treatment group demonstrated a significantly increased level of apoptosis when compared with that of the puerarin and 5-FU groups (P<0.01; Fig. 5).

### Evaluation of side effects

At the end of the study, blood was collected from the mice by cardiac puncture using heparin-rinsed 5-ml syringes and 22-gauge needles. The ALT, AST, BUN and Cr levels were measured to evaluate liver and renal injury (22). Serum ALT, AST, BUN and Cr levels were not identified to be significantly elevated in the treated mice when compared with those of the control group (P>0.05, Table III), and no significant difference in the levels of these biomarkers was identified between the puerarin, 5-FU and combined treatment groups (P>0.05, Table III). All mice were sacrificed and dissected following completion of the study. In addition, no liver or kidney injury, obvious metastasis or hemorrhage was observed. Furthermore, no significant difference was identified in liver or kidney weight between the treated groups and the control group, and no evident lesions were observed by histopathological examination.

## Discussion

HCC is a major public health threat that is responsible for ~600,000 mortalities each year (2,3). Recently, more effective treatments and earlier diagnosis have increased the survival rate. However, the efficacy of surgical techniques and radiotherapy is limited by the possibility of metastasis. Therefore, chemotherapy is key for improving survival in HCC patients.

5-FU is an important chemotherapeutic agent for the treatment of hepatogastroenteric tumors (23). 5-FU is commonly used to treat HCC, alone or in combination with other agents. Recently, more traditional medicines have been used to prevent or treat tumors, particularly in China. Puerarin has been used in traditional Chinese medicine for many years. Certain studies have suggested that puerarin exhibits anticancer activity and significant antiproliferative and apoptotic effects (9–16).

In the present study, the synergistic effect of puerarin and 5-FU treatment for hepatic carcinoma was investigated *in vitro* and *in vivo*. Puerarin or 5-FU alone were found to significantly inhibit the proliferation of SMMC7721 cells in a dose-dependent manner (puerarin, 400–6,400 μM; 5-FU, 40–640 μM). Furthermore, combined treatment with puerarin and 5-FU inhibited SMMC7721 cell proliferation at specific concentrations, when the fraction of affected cells was between 0.2555 and 0.7420. The mechanism of this synergistic effect was further investigated and the results showed that puerarin or 5-FU alone induced significant apoptosis when compared with that of the control group. In addition, the combined treatment induced a greater degree of apoptosis than puerarin or 5-FU alone *in vitro* and *in vivo*. This suggested that combined treatment with puerarin and 5-FU is more effective in inhibiting the growth of human HCC SMMC7721 cells.

A previous study suggested that puerarin may act as a sensitizer to enhance the inhibition of hepatic carcinoma cell proliferation by other chemotherapeutic agents (17). The results of the present study indicated that puerarin enhances the efficacy of 5-FU in HCC treatment *in vitro* and *in vivo*. Multiple factors have been implicated in the increased resistance to chemotherapeutic agents, including the reduction of intracellular drug accumulation and DNA damage repair by the modulation of proliferative or antiapoptotic proteins (24). Puerarin most likely enhances HCC sensitivity to 5-FU by inhibiting cell proliferation and inducing apoptosis.

Wang *et al* (11) showed that administration of puerarin was able to reverse multidrug resistance in a nude mouse model of human gastric carcinoma, and that decreased expression of P-glycoprotein and multidrug resistance-associated protein may be responsible for this effect. Crude pueraria extract and purified puerarin induce apoptosis, which may correlate with the observed inhibition of the cell cycle in the G0/G1 phase, as well as increased Bax expression and decreased Bcl-2 expression (13). In addition, Tran *et al* (15) revealed that puerarin downregulates multidrug resistance protein 1 expression in MCF-7/ADR cells via the upregulation of AMPK, which is dependent on nuclear factor-κ-β and cAMP-responsive element transcriptional activity. Further study is required to determine the molecular mechanism by which combined treatment with puerarin and 5-FU inhibits the growth of HCC. In conclusion, puerarin combined with 5-FU exhibited a significantly greater anti-liver cancer effect than that of puerarin or 5-FU treatment alone, and thus combined therapy may present a novel therapeutic regimen for HCC.

## Figures and Tables

**Table I:** Inhibitory effects of puerarin and 5-FU on SMMC7721 tumor volume in nude mice.

Group	n	Volume, mm^3^ (mean±SD)	Inhibition rate,%
Puerarin	6	134.89±45.71^a^,^b^	70.58
5-FU	6	108.83±12.93^a^,^b^	76.26
Puerarin + 5-FU	6	31.58±8.83^b^	93.11
Control	6	458.48±55.51	

Data are presented as inhibition rate (%) = (1 - mean of tumor volume from the experimental group/mean of tumor volume for the control) ×100 (n=6).

a P<0.05, vs. puerarin and 5-FU groups;

b P<0.05, vs. the control group.

5-FU, 5-fluorouracil.

**Table II:** Inhibitory effect of puerarin and 5-FU on SMMC7721 tumor weight in nude mice.

Group	n	Weight, g (mean±SD)	Inhibition rate,%
Puerarin	6	0.318±0.047)^a^,^b^	46.20
5-FU	6	0.297±0.068)^a^,^b^	49.86
Puerarin + 5-FU	6	0.147±0.029)^b^	75.21
Control	6	0.592±0.066)	

Data are presented as inhibition rate (%) = (1 - mean of tumor weight of tests/mean of tumor weight of control) ×100 (n=6).

a P<0.05, vs. puerarin + 5-FU group;

b P<0.05, vs. the control group.

5-FU, 5-fluorouracil.

**Table III:** Effect of puerarin combined with 5-FU or alone on hepatic and renal function.

Group	n	ALT, U/l (mean±SD)	AST, U/l) (mean±SD)	Urea, μmol/l) (mean±SD)	Cr, μmol/l) (mean±SD)
Puerarin	6	46.17±6.55	143.33±28.93	7.98±2.15	16.64±5.61
5-FU	6	46.00±3.16	144.83±21.27	7.96±0.96	15.86±5.29
Puerarin + 5-FU	6	45.33±8.16	146.67±25.84	8.29±1.59	16.24±3.54
Tumor control	6	46.33±4.08	145.33±14.85	8.05±0.51	16.57±4.54
Normal control	6	46.17±4.92	145.00±6.78	7.91±1.03	16.85±5.74

The groups were treated with puerarin (50 mg/kg/day), 5-FU (12 mg/kg/day), puerarin (50 mg/kg/day) and 5-FU (12 mg/kg/day), tumor control (an equal volume of saline) or normal control (no treatment). No differences in serum ALT, AST, urea or Cr were identified among the groups (P>0.05). 5-Fu, 5-fluorouracil; ALT, alanine aminotransferase; AST, aspartate aminotransferase; Cr, serum creatinine.
